# Conventional Dental Impressions vs. Impressions Reinforced with Rigid Mouthguards

**DOI:** 10.3390/polym16070994

**Published:** 2024-04-04

**Authors:** Andreea Codruta Novac, Anca Tudor, Daniela Maria Pop, Carina Sonia Neagu, Emanuela Lidia Crăciunescu, Mihai Romînu, Meda Lavinia Negruțiu, Virgil-Florin Duma, Cosmin Sinescu

**Affiliations:** 1 Faculty of Dental Medicine, “Victor Babes” University of Medicine and Pharmacy of Timisoara, 2 Eftimie Murgu Square, 300041 Timisoara, Romania; cojocariu.andreea@umft.ro (A.C.N.); popdanielamaria@yahoo.com (D.M.P.); carinaneagu2@gmail.com (C.S.N.); emanuela.craciunescu@umft.ro (E.L.C.); rominu.mihai@umft.ro (M.R.); negrutiu.meda@umft.ro (M.L.N.); minosinescu@yahoo.com (C.S.); 2 Research Center in Dental Medicine Using Conventional and Alternative Technologies, Department of Prostheses Technology and Dental Materials, Faculty of Dental Medicine, “Victor Babes” University of Medicine and Pharmacy of Timisoara, 9 Revolutiei 1989 Ave., 300070 Timisoara, Romania; 3 3OM Optomechatronics Group, Faculty of Engineering, “Aurel Vlaicu” University of Arad, 2 Elena Dragoi Str., 310177 Arad, Romania; 4 Faculty of Electronics, Telecommunications, and Information Technology, Polytechnic University of Timisoara, 2 Vasile Parvan Ave., 300223 Timisoara, Romania; 5 Center of Research and Development for Mechatronics, National University of Science and Technology POLITEHNICA Bucharest, Splaiul Independentei 313, Sector 6, 060042 Bucharest, Romania

**Keywords:** dental impressions, dimensional stability, polymeric materials, alginate, condensation silicone, addition silicone, mouthguard, reinforced dental impressions

## Abstract

The impression materials utilized today in dental medicine offer a good reproducibility and are easily accepted by patients. However, because they are polymer-based, they have issues regarding their dimensional stability. In this respect, the present work proposes a new type of dental impression, which is reinforced with rigid mouthguards. The aim of the study is to test the performances of such new impressions by comparing them to conventional ones—from this critical point of view, of the dimensional stability. Three types of polymeric materials were considered for both types of impressions: alginate, condensation silicone, and addition silicone. In order to obtain the new type of impressions, a manufacturing technique was developed, comprising the following phases: (i) conventional impressions were made; (ii) a plaster model was duplicated, and 15 rigid mouthguards were obtained; (iii) they were inserted in the impression technique, with each mouthguard positioned on the cast before the high-consistency material was inserted in the tray and the practitioner took the impression; (iv) the mouthguard remained in the tray and the low-viscosity material was inserted over the mouthguard; (v) the impression was positioned on the model, and after the material hardened, the mouthguard-reinforced impression was analyzed. In the evaluation of the dimensional stability, rigorous statistical analysis was essential to discern the performance differences between conventional and mouthguard-reinforced dental impressions. Statistical analyses employed non-parametric Mann–Whitney U tests because of the non-normal distribution of the data. They indicated a statistically significant improvement in the dimensional stability of addition silicone impressions when reinforced with mouthguards (*p* < 0.05), showcasing superior performance over conventional methods. Conversely, alginate and condensation silicone reinforced impressions did not exhibit the same level of stability improvement, suggesting the need for further optimization of these materials. In conclusion, from the three considered elastomers, addition silicone was found to be the prime candidate for high-precision dental impressions, with the potential to improve their quality from conventional impressions by utilizing the proposed reinforcing technique.

## 1. Introduction

A good prosthesis restoration depends on a variety of factors, which are present both in the clinical stages and in the technical laboratory. An important step is to obtain the dental impression. In this stage, the dentist must choose the most adequate impression material and technique [1,2]. If the medical doctor decides to take a conventional impression, she/he must choose between different materials, such as irreversible hydrocolloids or elastomers. However, optical impressions are popular today. They come in a large variety but not all dental practices have access to them. Also, there are limitations when it comes to optical impression systems that must still be overcome [3]. This is why traditional impressions are still the most common method. Generally speaking, dentists must have the ability to understand the advantages and drawbacks of each technology and impression technique in order to choose appropriately [3,4,5].

Impression materials can be classified according to their chemical composition, characteristic polymerization reaction, and setting time. Another classification can be made according to the material properties after setting [6]. Depending on these latter properties, impression materials can be classified as reversible or irreversible, as well as rigid (and semi-rigid) or elastic (Table 1) [7].

The problem when applying such materials pertains to the trade-off between their various (and often contradictory) characteristics. Thus, an ideal impression material would require properties that include good dimensional stability over time, reproducibility, elasticity, short setting time, enough working time, etc. [8,9,10,11,12]. The dimensional stability (i.e., their capacity to maintain their shape for a long period of time) is somewhat in contradiction to elasticity, but the latter is essential after the setting of the materials, making it possible to remove impressions from the oral cavity without suffering deformations [12]. When using such materials, one must also consider the fact that the best accuracy of the materials is after they polymerized; later on, it decreases in a variable way, depending on the material [9,10,11].

Therefore, the materials considered in this study are from the elastic impression materials, which can be divided into two groups: hydrocolloids and elastomers.

Of these materials, one of the most attractive is *alginate* (i.e., irreversible hydrocolloid), which is widely utilized in dental practice, despite its low dimensional stability. Alginate impressions are required during initial visits to the dental office in order to obtain a study model or document model. Extraoral (i.e., on the study model), after corroborating the data with clinical and radiological examinations, the doctor designs a treatment plan. Advantages of using alginate include the following: low price, ease of the impression technique (i.e., one impression at a time), pleasant taste and smell, easy acceptance by the patient, and minor discomfort because of the absence of the gag reflex [13,14].

*Elastomeric materials* include polysulfides, condensation and addition silicones, and polyether, as pointed out in Table 1.

*Polysulfides* belong to the category of synthetic rubbers and result from the polymerization of sulphureous hydrocarbons with a molecular weight of 300 to 400 at normal temperatures. They have the advantages of good reproducibility and tear resistance but have a series of drawbacks, including the fact that they are not easily accepted by the patient; therefore, they are not commonly utilized. On the other hand, polyether and silicones have a series of advantages, such as good elasticity, reproducibility of details, and good dimensional stability; these features make them more preferable to dentists [15,16,17,18,19,20]. Therefore, with their wide spectrum of indications, silicones and polyether are the most utilized impression materials in dental practice [21].

*Silicone elastomers* used for impressions are obtained either by polycondensation reaction or by polyaddition reaction; they are delivered in a two-component system, comprising a base and an activator. Thus, condensation silicones have as their base a polydimethylsiloxane and as their activator a suspension of tin octoate and ethyl orthosilicate; addition silicones have as their base a polyvinylsiloxane and as their activator a polysiloxane with a terminal vinyl group. Polyvinylsiloxanes (PVS) are impression materials with good dimensional stability; they are elastic, but hydrophobic. For this reason, the surface must be dry when taking the impression. Special attention must be paid in the case of subgingival preparations, for which the retraction cord highlights the preparation and prevents wetting with liquid from the gingival sulcus. Its presence increases the risk of defects in this area of the impression. The hydrophobic character of silicones is given to them by aliphatic hydrocarbon groups surrounding the siloxane bond [22]. Currently, there are PVS in which the manufacturers have added nonionic surfactants that make them less hydrophobic, resulting in vinylpolyethersiloxane (VPES) [23].

*Condensation silicones* have many advantageous qualities compared to PVS, including good dimensional stability, accuracy in rendering details, elasticity, and a lower price. Their polymerization reaction is carried out by linking the terminal groups of silicone polymer and alkyl silicate, forming a three-dimensional network. The by-product is ethyl alcohol; its evaporation can cause contraction of the material after setting [13]. On the other hand, PVS has a plugging reaction with the release of hydrogen in the form of gas, without secondary reaction by-products. For this reason, PVS are some of the most dimensionally stable impression materials [21].

*Polyethers* are hydrophilic elastomers that have dimensional stability and excellent detail reproduction. However, they are more rigid than silicones and therefore they can cause issues when disinserting from the oral cavity [24].

An overview of the different characteristic parameters of polymeric materials that are utilized or may be appropriate for dental impressions is provided in Appendix A.

Although they have better dimensional stability compared to alginates, the *polymerization contraction of elastomers has a negative effect on the dimensional stability and accuracy of this class of materials. This drawback that refers to dimensional stability characterizes all the above materials. It is inherent, as they are all polymers. This aspect can be a major issue in dental practice when it comes to obtaining good impressions.*

*Therefore, we have been looking for a solution, and this has defined the aim of this work: to* introduce a custom-fitted mouthguard to dental impression techniques and to study its effects. While mouthguards are usually utilized by patients with bruxism or as retainers in orthodontic treatments, to the best of our knowledge, this is the first time they have been considered in order to reinforce dental impressions.

The *Null hypothesis* of the study is that by using the newly proposed method (i.e., by introducing the mouthguards in dental impressions), there is no improvement in the dimensional stability of impressions, regardless of the utilized material.

In order to characterize this new method, the present study analyzes and compares improved mouthguard-reinforced dental impressions and conventional ones on the basis of dimensional stability, for three of the most utilized elastic impression materials, selected based on the discussion above.

## 2. Materials and Methods

### 2.1. Study Groups

In order to accomplish the proposed analysis, the three most common types of impression materials considered based on the comparison of their characteristics, advantages and drawbacks are alginate (Alligat, Kulzer, Hanau, Germany), condensation silicone (Polysiloxane, ZetaPlus, Zhermack, Italy), and addition silicone (Vinylpolysiloxane, Variotime, Kulzer, Hanau, Germany). Therefore, in order to compare the new proposed impression technique to the conventional one, six study groups were considered in this work, and they were as follows:

**Group 1 (CA)**—for conventional impressions made from alginate;
**Group 2 (CCS)**—for conventional impressions made from condensation silicone;
**Group 3 (CAS)**—for conventional impressions made from addition silicone;
**Group 4 (RA)**—for reinforced impressions made from alginate;
**Group 5 (RCS)**—for reinforced impressions made from condensation silicone;
**Group 6 (RAS)**—for reinforced impressions made from addition silicone.

For each material, five dental impressions were taken. Regarding this sample size, we conducted a G*Power test for *t* tests family—the Wilcoxon–Mann–Whitney test (two groups) with two tails, 90% power, 0.05 level of significance, and 0.6 effect size.

### 2.2. Sample Preparation

**Figure 1:**
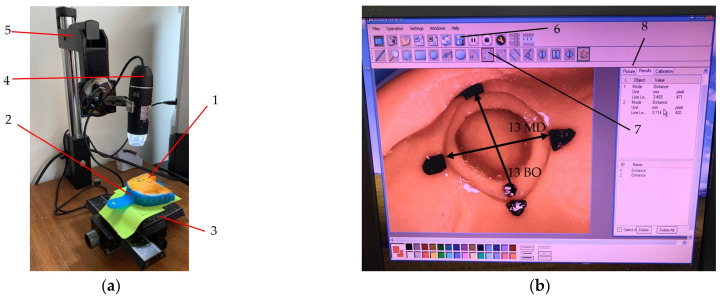
The measurement of a condensation silicone impression (1) in the standard impression tray (2): (**a**) dental impression positioned on (3) the sliding platform to allow for repeatability, with the position of the impression marked on the green paper; (**b**) the image captured by the camera (4) mounted on the system (5) for vertical adjustment of its position, as visualized on the screen. The image in (**b**) shows abutment 13 with markings for measuring both mesial-distal (MD) and buccal-oral (BO) distances. The button (6) for saving the images and the button (7) for measuring the distance between two points, while (8) shows the library with all saved images. Two distances are marked on the figure: the mesial-distal distance for the considered abutment (13 MD) and the buccal-oral distance for this abutment (13 BO).

The initial phase of this research involved selecting a plaster model featuring four abutments (16, 13, 23, and 26), each prepared with a chamfer finish line (Figure 1).

To mitigate the potential for cast damage throughout the study, this original model was replicated to produce six identical models. Subsequently, fifteen rigid mouthguards were fabricated based on these models. Each mouthguard was uniformly modified by cutting around the abutments epigingivally, connected by a 4 mm diameter strip. The underlying hypothesis posited that the mouthguard functions as a structural support, aiming to increase the dimensional stability of the impressions. The dimensional accuracy of these impressions was assessed using a digital microscope equipped with a camera (model SS-MS03, China). This microscope system (presented in Figure 1a) incorporates a high-quality CMOS color image sensor, which delivers a native resolution of 640 × 480 pixels and a maximum framerate of 30 fps. Its magnification range extends from 50× to 500×, which allows for a detailed examination of impression materials. The illumination is provided by eight white LEDs, equipped with a brightness control feature to improve image clarity under varying lighting conditions. For image capture and analysis, the utilized software was CoolingTech [25], compatible with Windows.

### 2.3. Measurement Protocols

Figure 1 illustrates the methodology for obtaining the measurements for a condensation silicone dental impression. Initially, the impression was placed on a designated measuring platform, with a digital camera capturing images of targeted areas. These images were then transferred to a computer for analysis, as depicted in Figure 1b, enabling precise measurement of the four abutments. For alginate impressions, a series of eight measurements were conducted within the first two hours post-impression, at 15 min intervals, as follows: t_1_ at minute 0; t_2_ at minute 15, t_3_ at minute 30, t_4_ at minute 45, t_5_ at minute 60, t_6_ after 1 h and 15 min., t_7_ after 1 h and 30 min., and t_8_ after 2 h.

In contrast, for elastomeric impressions, measurements were spaced out over the initial 24 h post-impression period, with the first measurement taken at the moment t_1_, exactly after the impression was taken; the second measuring moment t_2_ was taken after two hours, and the third moment t_3_ two hours after t_2_. These three moments were followed by five subsequent measurements (i.e., t_4_ to t_8_) every four hours until the eighth measurement.

**Figure 2:**
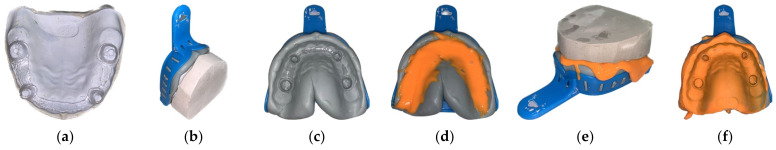
The impression procedure for the impressions obtained from condensation silicone using rigid mouthguards: (**a**) placing the mouthguard on the model; (**b**) the first step of the impression; (**c**) the putty material with the mouthguard in the impression; (**d**) the insertion of the fluid in the impression; (**e**) the second step of the impression; (**f**) the final impression.

**Figure 3:**
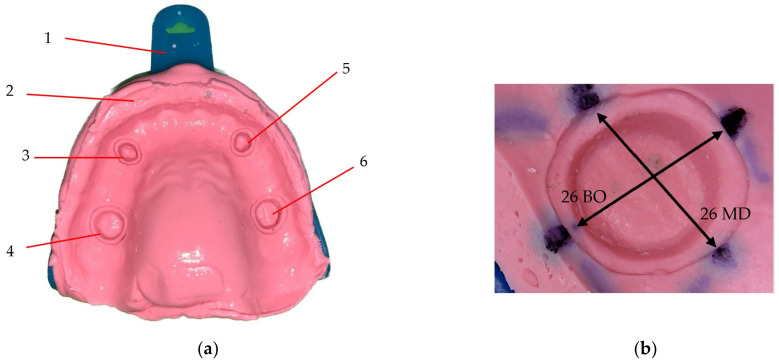
(**a**) Alginate impression with the mouthguard between the layers of the material, prepared for measuring the two distances on perpendicular directions for each abutment. Notations: (1) impression tray; (2) impression material; (3) impression of abutment 23; (4) impression of abutment 26; (5) impression of abutment 13; (6) impression of abutment 16. (**b**) Detailed image of the impression for abutment 26, with the MD and BO marked for measuring.

The protocol for the innovative impression technique utilizing a mouthguard comprises several critical steps (Figure 2): Initially, the mouthguard is positioned onto the model, followed by the introduction of the putty material into the tray for impression capture. Subsequently, once the material has solidified, the impression is carefully removed from the model. Notably, during this process, the mouthguard remains captured in the impression material, effectively becoming embedded within it. The next phase involves the preparation and application of a fluid material into the impression, emulating the conventional washing impression technique. This procedure ensures the mouthguard is encapsulated between the dual layers of impression materials (Figure 3).

Figure 2 delineates the procedural steps specific to the condensation silicone impression technique. Furthermore, the application of this technique to alginate impressions, incorporating the use of a mouthguard, adheres to a biphasic approach. In this case, the initial layer of alginate is formulated to exhibit high viscosity, whereas the subsequent layer is characterized by a reduced viscosity relative to the first, ensuring optimal material performance and impression accuracy.

All impressions were measured using the digital camera, as shown in Figure 1. In order to decrease the impact of errors, the position of the tray (2) (Figure 1a) was marked. Therefore, each impression (1) could be placed in the same position. Also, all the measuring points were marked on the impressions, as presented in Figure 1b. These aspects were a necessary measure in order to obtain an as high as possible reproducibility of the measurements. For each of the four abutments on an impression, the measurements were made in two perpendicular directions, i.e., mesial-distal (MD) and buccal-oral (BO), as presented in Figure 1b and Figure 3b.

### 2.4. Statistical Analysis

The statistical analysis was performed using SPSS v25. Quantitative variables were expressed as Mean ± Standard Deviation (SD) for normal distributed values, and as Median (Interquartile Range) for non-Gaussian distribution of data. The comparison was made using the non-parametric Mann–Whitney U Test (for the comparison between two groups) because the numerical data do not have a normal distribution (*p* < 0.001, Shapiro-Wilk Test). The results were considered significant for a value of *p* < 0.05.

## 3. Results

**Figure 4:**
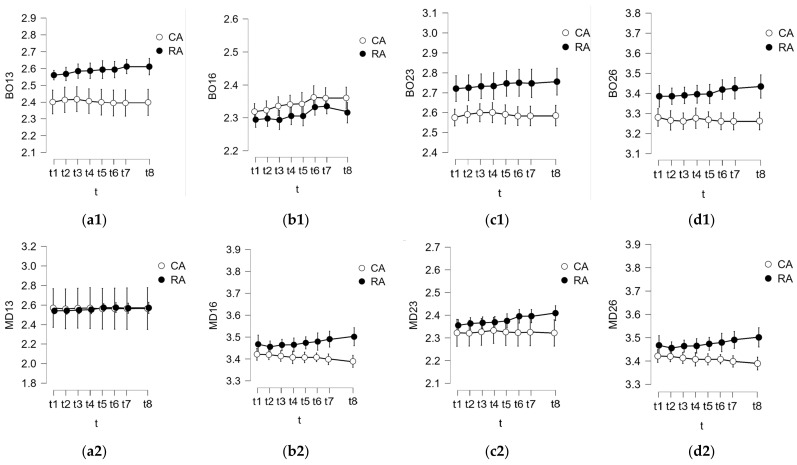
Results of the analysis performed at specific time moments for alginate regarding the comparison between conventional impressions (Group 1: CA) and mouthguard-reinforced impressions (Group 4: RA) for (**1**) BO and (**2**) MD, for the four considered abutments: (**a**) 13, (**b**) 16, (**c**) 23, and (**d**) 26.

**Figure 5:**
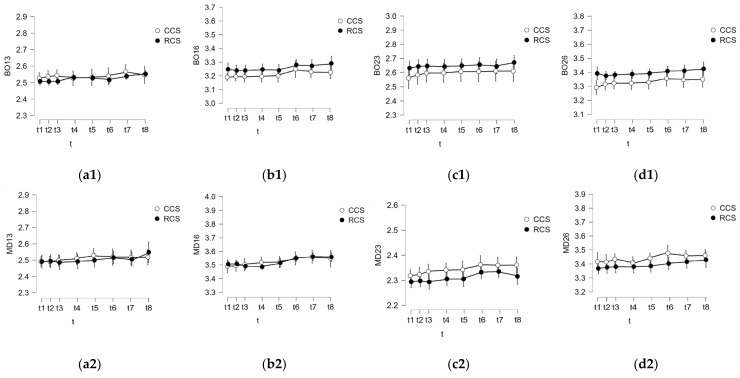
Results of the analysis performed at specific time moments for condensation silicone regarding the comparison between conventional impressions (Group 2: CCS) and mouthguard-reinforced impressions (Group 4: RCS) for (**1**) BO and (**2**) MD, for the abutments (**a**) 13, (**b**) 16, (**c**) 23, and (**d**) 26.

**Figure 6:**
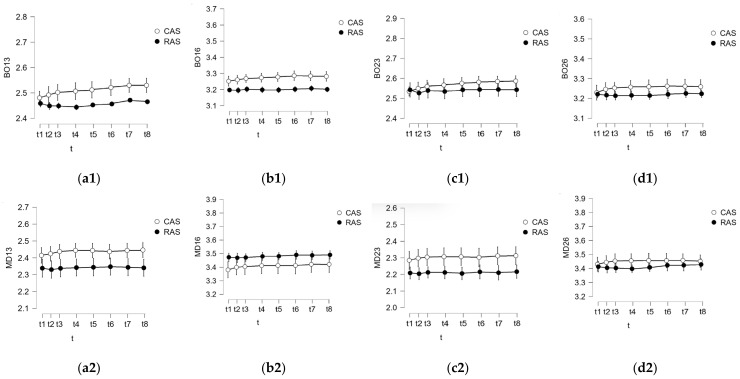
Results of the analysis performed at specific time moments for addition silicone regarding the comparison between conventional impressions (Group 3: CAS) and mouthguard-reinforced impressions (Group 5: RAS) for (**1**) BO and (**2**) MD, for the abutments (**a**) 13, (**b**) 16, (**c**) 23, and (**d**) 26.

During the initial stage, data analyses were conducted. We scrutinized measurements taken at the considered time moments t_k_, k = $\;\bar{1.8}$. Both the mean and standard deviation (SD) were computed for the entire dataset spanning these eight moments. Figure 4, Figure 5 and Figure 6 show these means and SDs from t_1_ to t_8_ for the six data sets obtained for both BO and MD measured distances (corresponding to the six study groups). In order to facilitate an easier comparison between the conventional and mouthguard-reinforced impressions, both means and SDs were grouped for each type of measurement (CA with RA, CCS with RCS, CAS with RAS) for each of the three considered materials, in each corresponding figure, from Figure 4, Figure 5 and Figure 6.

A comparative analysis was conducted to evaluate the deformations between conventional impressions (Groups 1 to 3, i.e., CA, CCS, and CAS) and mouthguard-reinforced impressions (Groups 4 to 6, i.e., RA, RCS, and RAS) for each of the considered materials. For all the buccal-oral (BO) and mesial-distal (MD) measured distances, the analyses involved two series, each comprising 40 values (N = 40 for both cases), facilitating a comprehensive comparison.

The outcomes of these analyses, detailing the comparison between conventional and reinforced impressions for the four abutments, are systematically presented in Table 2 for alginate, Table 3 for condensation silicone, and Table 4 for addition silicone impressions. A low *p*-value (typically smaller than 0.05) suggests that there is a statistically significant difference between the RA and CA groups for that variable. As all variables in Table 2 show a *p*-value < 0.05, this indicates a statistically significant difference between the RA and CA groups for each considered variable (i.e., measured dimension). For all variables, the median values for RA are higher than those for CA, suggesting that RA does not improve the impression technique in terms of reducing dimensional changes. Therefore, based on this analysis, RA does not represent an improvement over CA in terms of minimizing dimensional changes. A similar conclusion can be extracted following the comparison between the RCS and the CCS performed in Table 3.

When interpreting the results from Table 4, the same threshold of 0.05 was considered for the *p*-value to indicate the potentially statistically significant difference between RAS and CAS. Therefore, there are significant differences for all measurements, except 16MD. This suggests that there is a statistically significant improvement in the measured dimension for RAS compared to CAS.

The comparison of means for each performed measurement between RAS and CAS shows that for 13BO, 13MD, 16BO, 23BO, 23MD, 26BO, and 26MD, the RAS group had lower mean values compared to the CAS group, indicating that it performed better (i.e., with a lower dimensional change). MD16 was the only measurement where RAS had a higher mean value than CAS, suggesting that in this particular case, RAS might not be better than CAS. In summary, for the majority of the measurements, RAS demonstrates an improvement over CAS by displaying lower dimensional changes.

**Figure 7:**
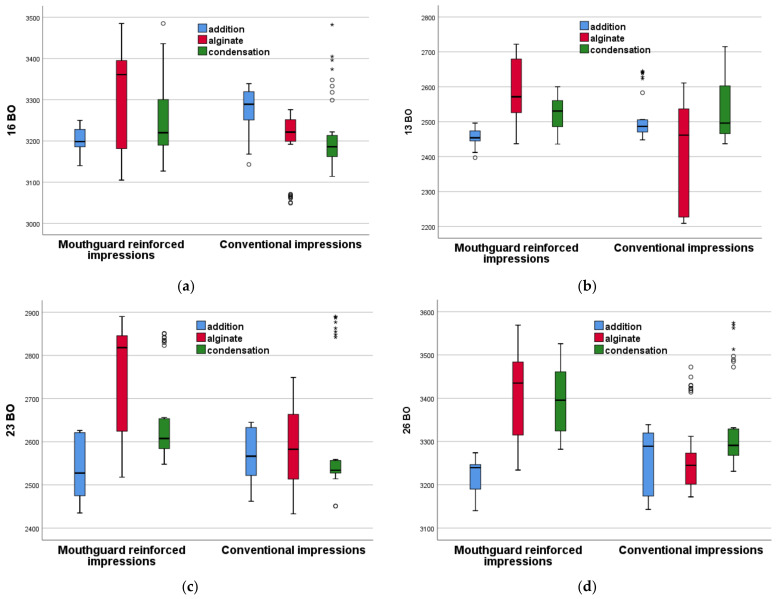
Boxplots for the mouthguard-reinforced impressions compared to conventional impressions for each of the three considered materials, for: (**a**) 16 BO, (**b**) 13 BO, (**c**) 23 BO, and (**d**) 26 BO. Explanations of the notations are made in the text.

**Figure 8:**
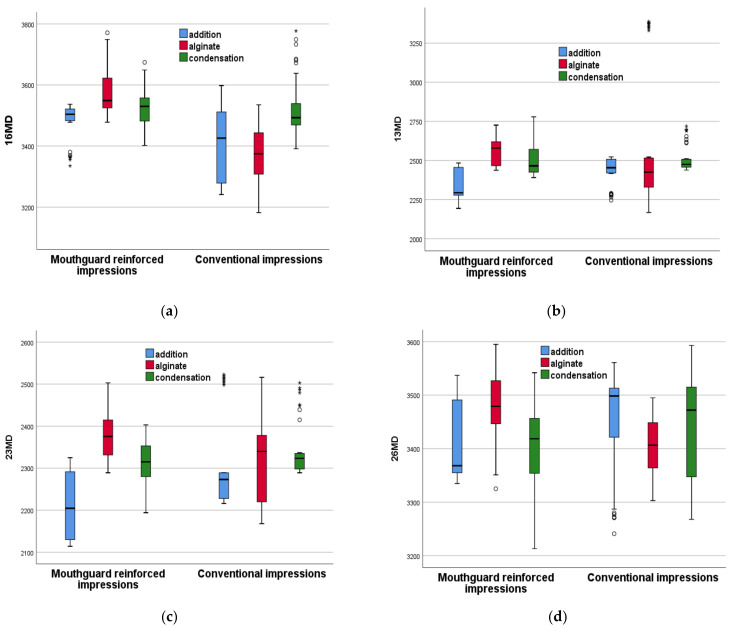
Boxplots for the mouthguard-reinforced impressions compared to conventional impressions for each of the three considered materials, for: (**a**) 16 MD, (**b**) 13 MD, (**c**) 23 MD, and (**d**) 26 MD. Explanations of the notations are made in the text.

Figure 7 and Figure 8 show the novel mouthguard-reinforced impressions compared to conventional impressions for each of the three considered materials in a more synthetic way. Each boxplot represents the distribution of the measurements for every type of impression material in the study: **addition silicone** (represented by the blue boxplots), **condensation silicone** (red boxplots), and **alginate** (green boxplots).

For each boxplot, the **central line** in the box represents the median of the data, the **box itself** shows the interquartile range (IQR), and the **whiskers** (lines extending from the top and bottom of the box) represent the range of the data, which typically extends to 1.5 times the I^QR^ above the 75th percentile and below the 25th percentile. Data points beyond the whiskers are considered outliers; the **circles** outside of the whiskers are the outliers, which are data points that fall beyond the range of what is considered normal variability (i.e., 1.5 times the IQR); the **stars** representdata points identified as extremes, which signify observations lying outside the typical range. These notations facilitate the distinction of values that significantly deviate from the rest of the data set, thus enabling a more comprehensive analysis of data dispersion and variability.

The boxplots allow for a comparison between the different materials with regards to their central tendency (median), variability (spread of the IQR), and the presence of outliers, which can provide insights into the stability and consistency of the impression materials. This representation confirms the statistical analysis above, and it also allows for comparison among the different groups. Thus, one may conclude that, *for conventional impressions, none of the three considered materials is superior to the other two because for the eight measured dimensions, some are higher for a certain material*; there is no consistency.

However, for the novel proposed method of reinforced impressions, addition silicone has consistently lower values compared to the other two materials. This result can be coupled with the one found in Table 4, which demonstrates that from seven out of eight evaluated dimensions, the RAS group has an improvement over the CAS group. This allows for the determination of the best material out of the three considered in the study. Also, this demonstrates an improvement obtained using the novel impressions with regard to the conventional ones—if a proper material is chosen.

## 4. Discussion

Elastomeric materials are predominantly favored by dental professionals for impressions in fixed prosthodontics because of their dimensional stability, which is crucial for reaching accurate outcomes. The properties of impression materials, including their dimensional stability, have been extensively investigated in the literature [2,6,7]. The present study showed that, by introducing a hard skeleton made of a mouthguard between the two layers of the utilized polymeric material, the dimensional stability of the impression could be improved if appropriate materials are employed.

### 4.1. Comparison between the Different Groups

(i) In the comparative analysis of reinforced alginate (RA) and conventional alginate (CA) impressions, RA demonstrated statistically significant differences in all measured variables (Table 2, Figure 7 and Figure 8). However, these differences indicated increased dimensional changes in RA compared to CA, as pointed out by higher median values across all measurement parameters.

While at first glance this may suggest a disadvantage for RA, it is important to consider these findings within a broader context. The increased dimensional changes associated with RA do not necessarily negate its potential utility. Factors such as an improved material strength, ease of handling, or longer-lasting impressions may still make RA a valuable choice in specific clinical scenarios. Further research is warranted to explore these aspects and to understand the underlying mechanisms contributing to the observed dimensional changes.

Moreover, this study opens avenues for the refinement of RA materials. Understanding the factors leading to increased dimensional changes can guide the development of improved RA formulations. Future iterations of RA might maintain its advantageous properties, while mitigating the increased dimensional changes observed in this study.

(ii) In the comparative analysis between reinforced condensation silicone (RCS) and conventional condensation silicone (CCS), statistically significant differences were observed in specific measurement variables (Table 3, Figure 7 and Figure 8). Notably, RCS demonstrated improved results in the MD26 measurement, indicating potential advantages in certain aspects of the dimensional stability. However, in other parameters such as BO16, BO23, and BO26, RCS did not exhibit superior performance compared to the conventional method.

These findings suggest that while RCS has not uniformly outperformed CCS in reducing dimensional changes, its enhanced performance in certain areas highlights its potential utility. The observed improvements in specific measurements are promising and warrant further investigation. They point to the possibility that with additional refinement and optimization, the novel impression method could offer advantages when using existing condensation silicone impression materials.

It is also important to consider that the utility of RCS may extend beyond the scope of this study. Factors such as material durability, ease of handling, and patient comfort, which were not the focus of our current analysis, could significantly contribute to the overall value of RCS in clinical settings. Future research should aim to not only address the areas where RCS currently falls short but also explore its full range of properties. Such comprehensive investigations will be important in determining the practical applicability and potential superiority of RCS over conventional materials in dental impression technology.

(iii) In the comparative analysis of reinforced addition silicone (RAS) and conventional addition silicone (CAS) impressions, RAS exhibited significant potential for enhancing impression accuracy and stability (Table 4, Figure 7 and Figure 8). The statistical analysis, which specifically employed the Mann–Whitney U test, indicated that RAS consistently yields lower dimensional changes across most of the measured parameters (i.e., BO13, MD13, BO16, BO23, MD23, BO26, and MD26), with *p*-values signalling strong statistical significance (i.e., *p* < 0.05). Given that increased dimensional changes are deemed detrimental for the precision of dental impressions, the observed reduction in these changes with RAS highlights its superior performance in maintaining dimensional integrity.

The mean values for measurements such as BO13, MD13, and BO23, among others, were significantly lower for RAS compared to CAS, suggesting a notable improvement in minimizing dimensional variability, a critical factor in the fidelity of dental impressions. Only in the case of MD16 did RAS not outperform CAS, which points to the necessity for further investigation into specific conditions or parameters under which RAS might exhibit variable outcomes.

These results underscore RAS’s potential to significantly reduce dimensional changes associated with conventional addition silicone impressions, thereby increasing the accuracy and reliability of dental impressions. This potential improvement is not only statistically significant but also bears clinical relevance, as even minor deviations in impression accuracy can lead to substantial discrepancies in the final dental restoration. Therefore, RAS emerges as a promising alternative to conventional addition silicone impression techniques, warranting further research and development to fully harness its advantages for clinical applications.

### 4.2. Defects in the Obtained Impressions

Because alginates and elastomers have a different dimensional stability in time, we chose to analyze alginates for a shorter period of time compared to elastomers. The dental technician knows that the alginate has to be preserved in an environment with 100% humidity until the cast is made. In this respect, a further study is planned in order to analyze the period of dimensional stability for alginate impressions in such a 100% humidity environment.

Among the evaluated materials, the alginate impressions are not considered to have the precision and dimensional stability necessary for them to be used in fixed prostheses treatments [26,27,28,29,30]. However, they were used in the present study in order to assess their eventual possibility to be utilized using the novel, proposed technique.

**Figure 9:**
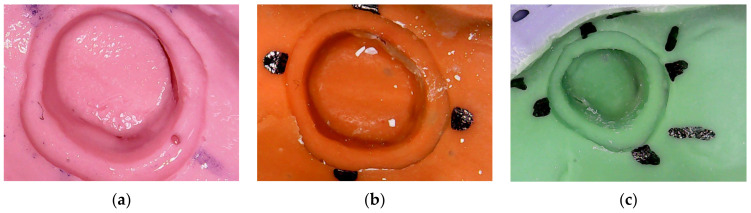
Defects in impressions using the mouthguard, for the three considered polymeric materials: (**a**) alginate; (**b**) condensation silicone; (**c**) addition silicone.

After comparing conventional impressions to mouthguard impressions, the first difference we observed was in the appearance of defects in the abutment areas in some of the mouthguard impressions. These were produced by the friction between the material and the plaster when disinserting the impression from the model. Therefore, a tearing of the impression material occurred, as can be noticed in Figure 9. This phenomenon is caused by two factors: the layer of material inside the mouthguard being very thin and the plaster model having a certain roughness. It must be mentioned that an impression in the oral cavity might not present the same defects. The mouthguard had an orifice in the occlusal area of each abutment in order to link the two layers of material and thus, maintain a good dimensional stability in the low-viscosity material. Furthermore, it was important to cut the mouthguard at epigingival level in the area of the finish line of the preparation, in order to obtain a link between the putty material and the low viscosity material. Therefore, a good stability in the fluid in that area could be obtained.

Besides the dimensional stability of the impressions, defects in the materials have an impact on the quality of the adaptation of the future prosthetic restoration. The results of several studies have shown an improvement in the accuracy and properties of today’s impression materials [31]. The quality of conventional dental impressions that were received by dental laboratories for manufacturing fixed prosthetic restorations have clearly remained inadequate [31,32]. An early 1997 study analyzed 290 impressions in 4 commercial dental laboratories; it was observed that 36% of the examined impressions presented errors [33]. In 1999, another study showed that 50% of the impressions sent to the laboratories had errors [34]. Furthermore, in 2005, an analysis of 193 impressions from 11 dental laboratories concluded that 89% of the evaluated impressions presented at least 1 error [31]. In 2014, 200 impressions from 4 laboratories in Malaysia were studied; the results showed that 64.5% of the impressions were inadequate [35]. In 2017, a study from North Carolina concluded that 86% of the impressions evaluated presented at least 1 error; 55% of the defects were in the finish line of the preparation area, which is critical for the adaptation of the restoration [35]. Therefore, after several decades of examining this problem, we can conclude that it still creates major issues for practitioners.

In contrast, in our study, using the proposed reinforced design, the defects in the material were on the surface of the abutment, not in the finish line area. However, a satisfactory impression must not have defects at all, in order to obtain a good adaptation of the future restoration on the abutment. Therefore, we envisage future work in order to be able to tackle this issue as well.

### 4.3. Discussion Regarding Different Impressions Materials

In studies that have analyzed the dimensional stability of the three considered elastomers (i.e., the condensation and addition silicones, as well as the polyether), it was concluded that in the first 0.5 h, all materials showed a satisfactory dimensional stability [36,37,38,39,40,41]. The same result was obtained in our study, for the two elastomers. Abd-Al Hamed et al. analyzed the dimensional stability of elastomers using a 3D laser scanner and showed that the addition silicone had an appropriate dimensional stability [12]. In our study, the material with the best dimensional stability was the addition silicone as well, even after using the novel reinforcement technique that we introduced.

The present study started from the hypothesis that mouthguards act like a skeleton, providing rigidity to the impression materials. Thus, mouthguards can increase their dimensional stability.

(i) It was assumed that alginate, as a material that undergoes large volumetric changes over time, would show considerable improvements with the introduction of the mouthguards. However, the opposite result was obtained. A reason for this result may be the small thickness of the material layer at the interface between the model and the mouthguard. Because alginate is prone to dehydration in room-temperature environments, the thin layer of material at the level of the abutments suffered larger dimensional variations. It is also known that alginate does not have satisfactory properties for impression in fixed prosthetics [26,27,28,29,30]. Thus, this study agrees with the literature regarding alginate’s lack of dimensional stability and reproducibility, as well as the precision and fidelity necessary for fixed prosthetic restorations. This material has its uses: impressions for orthodontics, duplicates, document and study models, or mobile prosthodontics [26,27,28,29,30]. Also, it must be mentioned that the impressions were not kept in 100% humidity conditions. This indicates the need for future work, as explained above.

(ii) Condensation silicones are materials that are utilized in fixed prosthetics. As a result of the measurements performed in this study, no improvements in their dimensional stability were observed, even when introducing the mouthguards in the impression technique. This result can be explained by the fact that the mouthguard is interposed between the putty and the light body material. It is well known that the light body condensation silicone has a larger dimensional variation compared to the putty material; as it is isolated from it, contractions and expansions are allowed [4].

(iii) The best results were obtained in this work using the addition silicone. The improvement in the dimensional stability showed that introducing mouthguards in the impression technique for such materials significantly increases the fidelity and precision of dental impressions. By comparing the average error obtained for the dimensional variations of addition and condensation silicone impressions, an increase was observed in the dimensional variations of condensation silicone impressions. This demonstrated the superiority of additional silicone for this dental medicine application.

### 4.4. Discussion Regarding the Impression Technique

Torassian et al. showed that there is a connection between the technique by which the impression is made and the time until the model is cast [42]. Thus, for each individual material, the casting of the model must be done on time.

(i) For alginate impressions, Bud et al. [43] recommended casting the model 10–12 min. after the disinsertion from the oral cavity. This is particularly difficult to achieve in situations where the dental laboratory is not in the same facility as the dental practice. The low dimensional stability of alginate is produced by the phenomena of imbibition and syneresis [44,45]. Also, Peutzfeld et al. observed that out of all the studied materials, alginate had the lowest accuracy [46].

Certain studies tried to improve the alginate formula by adding silicon lead or cadmium. Studies have shown that this procedure can increase the time for casting the model by up to 4 weeks, but the toxicity of cadmium must be carefully considered [47,48].

Regarding the defects in alginate impressions, Cervino et al. [13] showed that they can be improved by smoothing the material loaded in the impression tray with a wet finger before inserting it into the oral cavity. For our study, the material was smoothed with a spatula when it was inserted into the impression tray. The same author recommended rinsing the patient’s oral cavity with water just before the impression, in order to decrease the concentration of saliva that comes in contact with the impression material [13].

(ii) When it comes to condensation silicones, an issue they have is hydrophobicity. This is due to the formation of a chain of surface paraffin groups. In situations where the surface is not dried before the impression procedure, the liquid particles (saliva or blood) are incorporated on the surface of the material [11,49,50]. There have been attempts to improve the formula of materials by adding nonionic surfactants. It was observed that they detach from the superficial surface of the impression when it is disinfected or washed under a strong jet of water. This happens because of ethyl alcohol, which results as a by-product of the polymerization reaction of condensation silicone, which is a small, plasticizing molecule [51]. The reaction by-product after setting leads to the contraction of the material, through the evaporation of the alcohol over time, resulting in a decrease in dimensional stability and accuracy.

(iii) For addition silicone, a better stability is observed. Because it has no polymerization by-products, its dimensional stability and accuracy are maintained for a longer period of time. Thus, Martins et al. concluded that addition silicone can be stored for a week until the casting of the model. The dimensional variations are minimal and do not influence the clinical results [11].

The impression technique is an important factor that influences both accuracy and precision [1,4]. Besides this, impression trays play an important role [5]. Nouri et al. observed that rigid impression trays represent a good alternative to plastic ones because the latter can be deformed during the impression and disinsertion from the oral cavity [52]. When comparing standard rigid impression trays and individual ones, no considerable improvement was observed when using the latter with regards to dimensional stability and the accuracy of the impressions.

Lee et al. observed that digitalization improves the quality of patients’ experience and eases the work of dentists during the treatment [53]. However, the accuracy of digital impressions is not better than that of conventional ones [54,55]. The literature generally suggests that digital impressions offer several advantages over conventional impressions, including efficiency and patient comfort. Digital techniques reduce the potential for errors associated with the physical impression materials and processes. Also, they allow for immediate review and adjustment, potentially saving time and resources. However, the success of digital impressions can depend on the specific technology utilized and on the operator’s proficiency. Despite these benefits, some studies note that the initial cost of digital systems can be high, and there may be a learning curve associated with their adoption [56,57,58,59,60,61,62,63,64,65].

Finally, by analyzing the literature regarding impression materials, Naumovski et al. have observed that until now, addition silicone is superior to the others due to its advantages [66], which is in agreement with the results obtained in this work.

In the present study, improvements made by introducing mouthguards in the impression technique include the following: (a) the impression technique is not more difficult; (b) although this technique involves new steps in taking a first impression, casting the model and creating the mouthguard, it has certain benefits because the dentist can visualize and analyze the preparations on the model, and observe if the abutments are parallel, if there are any areas that need to be corrected, and if there is enough space for the restorations; (c) the cost of introducing this novel, proposed step is minimal.

Limitations of the present work must also be highlighted. This is a preliminary, in vitro study. There may be differences between the conditions encountered clinically and those in vitro, which we did not take into consideration. However, they may influence results in a dental office. Also, the number of samples was limited. Therefore, future research in this field must be carried out.

## 5. Conclusions

The present study proposed a novel method for improving the dimensional stability of dental impressions by introducing rigid mouthguards in their manufacturing process. The performances of such new impressions were compared to conventional ones. For both types of impressions, three types of polymeric materials were considered: alginate, condensation silicone, and addition silicone. While mouthguard-reinforced alginate impressions (RA) did not demonstrate superiority over conventional impressions (CA) in terms of dimensional stability, their potential advantages in other areas coupled with opportunities for material improvement highlighted their ongoing relevance and promise in dental impression technology. Continued research and development are needed to fully realize the potential of mouthguard reinforced alginate impressions. Also, the mouthguard reinforced condensation silicone impressions (RCS) have yet to demonstrate conclusive superiority in all aspects of dimensional stability. Continued research, development, and refinement are imperative to fully harness the capabilities of the novel technique regarding this material as well. Therefore, future work is planned to explore the possibilities for improving the dimensional stability of dental impressions manufactured using these two types of (most common) polymeric materials and the novel proposed method and to fully exploit their advantages, including the degree of acceptance from patients.

Regarding the third considered material, the findings of this study succeeded in highlighting the considerable promise of reinforced addition silicone impressions (RAS) in reducing the dimensional changes commonly associated with conventional addition silicone impressions (CAS), thereby potentially elevating both the precision and reliability of dental impressions. Given the critical impact of even minimal inaccuracies in impression dimensions on the fidelity of the final dental restorations, the capability of RAS to consistently minimize such variability can be a noteworthy advancement in dental impression technology. Consequently, RAS positions itself as a promising innovation in the realm of addition silicone impression techniques, necessitating further exploration and optimization to fully exploit its clinical benefits.

In conclusion, the null hypothesis of the study was rejected, as the work demonstrated that by using the new proposed method that includes mouthguards in dental impressions, an improvement in the dimensional stability of impressions can be obtained for certain materials.

## Figures and Tables

**Table 1:** Classification of dental impression materials—an overview.

Materials	Rigid and Semi-Rigid	Elastics
**Irreversible**	Gypsum Acrylic Resin Zinc Oxide Eugenol paste	Alginates Polysulfides Polyethers Silicones (condensation or addition)
**Reversible**	Waxes Thermoplastic materials Bucco-plastic materials Gutta-percha	Agar-agar hydrocolloids

**Table 2:** Descriptive statistics and *p* values of the conventional vs. reinforced alginate impressions for the four abutments with BO and MD measurements: Group 1 (CA) vs. 4 (RA).

Tooth		Conventional Impressions: Group 1 (CA)		Mouthguard-Reinforced Impressions: Group 4 (RA)		*p* M–W
		Mean ± SD (μm)	Median (Interquartile Range) (μm)	Mean ± SD (μm)	Median (Interquartile Range) (μ)	
*16*	BO	3199.9 ± 73.38	3221.5 (53)	3299.1 ± 120.46	3361 (216)	0.005
	MD	3363.5 ± 98.09	3374.5 (137)	3575.3 ± 75.96	3549 (106)	<0.001
*13*	BO	2403.1 ± 154.34	2461.5 (313)	2588.9 ± 91.1	2571.5 (155)	<0.001
	MD	2565.9 ± 419.36	2426 (189)	2569.6 ± 90.14	2579.5 (153)	0.001
*23*	BO	2588.2 ± 95.83	2582.5 (156)	2739.5 ± 133.99	2818 (223)	<0.001
	MD	2324.9 ± 118.77	2340 (160)	2378.8 ± 60.18	2375.5 (90)	0.026
*26*	BO	3267.4 ± 89.82	3245 (74)	3404.5 ± 00.48	3435 (175)	<0.001
	MD	3407.9 ± 51.55	3406.5 (87)	3474.6 ± 70.9	3479 (85)	<0.001

Mann–Whitney (M–W) U Test; data are represented as mean ± SD and as median (interquartile range).

**Table 3:** Descriptive statistics and *p* values of the conventional vs. reinforced condensation silicone impressions for the four abutments with BO and MD measurements: Group 2 (CCS) vs. 4 (RCS).

Tooth		Conventional Impressions: Group 2 (CCS)		Mouthguard-Reinforced Impressions: Group 5 (RCS)		*p* M–W
		Mean ± SD (μm)	Median (Interquartile Range) (μm)	Mean ± SD (μm)	Median (Interquartile Range) (μm)	
*16*	BO	3209.8 ± 89.14	3186 (56)	3257 ± 89.86	3220 (114)	0.003
	MD	3523.2 ± 98.33	3493 (71)	3522.3 ± 59.87	3530 (80)	0.227
*13*	BO	2539.2 ± 88.79	2496 (140)	2523.4 ± 43.14	2530.5 (76)	0.988
	MD	2509.3 ± 82.9	2475.5 (53)	2504.1 ± 92.88	2466 (151)	0.290
*23*	BO	2596.3 ± 139.86	2534 (31)	2647.6 ± 100.98	2607.5 (73)	<0.001
	MD	2343.1 ± 64.48	2323.5 (39)	2309.9 ± 55.85	2315 (74)	0.218
*26*	BO	3329.2 ± 102	3291 (64)	3397.5 ± 75.98	3395.5 (140)	<0.001
	MD	3438.4 ± 98.21	3472 (168)	3392.8 ± 94.93	3418.5 (104)	0.065

Mann–Whitney (M–W) U Test; data are represented as mean ± SD and as median (interquartile range).

**Table 4:** Descriptive statistics and *p* values of the conventional vs. reinforced addition silicone impressions for the four abutments with the BO and MD measurements: Group 3 (CAS) vs. 6 (RAS).

Tooth		Conventional Impressions: Group 3 (CAS)		Mouthguard-Reinforced Impressions: Group 6 (RAS)		*p* M–W
		Mean ± SD (μm)	Median (Interquartile Range) (μm)	Mean ± SD (μm)	Median (Interquartile Range) (μm)	
*16*	BO	3273.3 ± 57.95	3289 (71)	3200.3 ± 32.8	3198.5 (43)	<0.001
	MD	3408.4 ± 120.81	3426 (234)	3479.7 ± 61.87	3504 (38)	0.055
*13*	BO	2509.2 ± 63.77	2486.5 (36)	2455.6 ± 21.51	2454 (30)	<0.001
	MD	2436.6 ± 87.35	2453.5 (90)	2340.8 ± 105.22	2293.5 (181)	0.001
*23*	BO	2567.5 ± 60.32	2566.5 (114)	2539.9 ± 71.05	2527.5 (149)	0.022
	MD	2303.5 ± 107.95	2273 (62)	2210.4 ± 77.83	2204.5 (162)	0.003
*26*	BO	3253.2 ± 71.51	3289 (146)	3218.8 ± 42.12	3239.5 (58)	0.019
	MD	3451.5 ± 98.82	3498.5 (92)	3413.3 ± 72.94	3368 (137)	0.017

Mann–Whitney (M–W) U Test; data are represented as mean ± SD and as median (interquartile range).

## Data Availability

The data presented in this study are available on request from the corresponding author.

## Appendix A

**Table A1:** Overview of characteristic parameters of polymeric materials that are utilized or may be appropriate for dental impressions.

Polymeric Material	Consistency	Residual Deformation (%)	Flexibility (%)	*Dimensional Variation (%)*	Hardness Shore A	Tear Resistance (g/cm)
**Polysulfides**	low	3–4	14–17	0.5–2	20	2500–7000
	medium	3–5	11–15	0.5–1	30	3000–7000
	heavy	3–6	9–12	0.5–1	35	-
**Polyether**	low	1.5	3	0.03	35–40	1800
	medium	1–2	2–3	0.02	35–60	2800–4800
	Medium + viscosity-lowering agent	2	6	0.04	30–50	2500
	Heavy	2	3	0.02	40–50	3000
**Condensation silicones**	low	1–2	4–9	0.05–0.1	15–30	2300–2600
	putty	2–3	2–5	0.02–0.05	50–65	-
**Addition silicones**	low	0.05–0.4	3–6	0.01–0.03	35	1500–3000
	medium	0.05–0.3	2–5	0.01–0.03	50	2200–3500
	heavy	0.1–0.3	2–3	0.01–0.03	60	2500–4300
	putty	0.2–0.5	1–2	0.01–0.1	50–75	-
